# Temporal dynamics of fecal microbiota community succession in broiler chickens, calves, and piglets under aerobic exposure

**DOI:** 10.1128/spectrum.04084-23

**Published:** 2024-05-08

**Authors:** Paul Oladele, Jennifer Ngo, Tiffany Chang, Timothy A. Johnson

**Affiliations:** 1 Department of Animal Sciences, Purdue University, West Lafayette, Indiana, USA; Dominican University New York, Orangeburg, New York, USA

**Keywords:** fecal microbiome, broiler, calf, piglet, 16S rRNA, alpha diversity, beta diversity, random forest

## Abstract

Researchers have extensively studied the effect of oxygen on the growth and survival of bacteria. However, the impact of oxygen on bacterial community structure, particularly its ability to select for taxa within the context of a complex microbial community, is still unclear. In a 21-day microcosm experiment, we investigated the effect of aerobic exposure on the fecal community structure and succession pattern in broiler, calf, and piglet feces (*n* = 10 for each feces type). Bacterial diversity decreased and community structure changed rapidly in the broiler microbiome (*P* < 0.001), while the fecal community of calves and piglets, which have higher initial diversity, was stable after initial exposure but decreased in diversity after 3 days (*P* < 0.001). The response to aerobic exposure was host animal specific, but in all three animals, the change in community structure was driven by a decrease in anaerobic species, primarily belonging to Firmicutes and Bacteroidetes (except in broilers where Bacteroidetes increased), along with an increase in aerobic species belonging to Proteobacteria and Actinobacteria. Using random forest regression, we identified microbial features that predict aerobic exposure. In all three animals, host-beneficial *Prevotella*-related ASVs decreased after exposure, while ASVs belonging to *Acinetobacter*, *Corynbacterium,* and *Tissierella* were increased. The decrease of *Prevotella* was rapid in broilers but delayed in calves and piglets. Knowing when these pathobionts increase in abundance after aerobic exposure could inform farm sanitation practices and could be important in designing animal experiments that modulate the microbiome.

**IMPORTANCE:**

The fecal microbial community is contained within a dynamic ecosystem of interacting microbes that varies in biotic and abiotic components across different animal species. Although oxygen affects bacterial growth, its specific impact on the structure of complex communities, such as those found in feces, and how these effects vary between different animal species are poorly understood. In this study, we demonstrate that the effect of aerobic exposure on the fecal microbiota was host-animal-specific, primarily driven by a decrease in Firmicutes and Bacteroidetes, but accompanied by an increase in Actinobacteria, Proteobacteria, and other pathobionts. Interestingly, we observed that more complex communities from pig and cattle exhibited initial resilience, while a less diverse community from broilers displayed a rapid response to aerobic exposure. Our findings offer insights that can inform farm sanitation practices, as well as experimental design, sample collection, and processing protocols for microbiome studies across various animal species.

## INTRODUCTION

Animal production systems have become more consolidated into larger operations, leading to the generation of more manure. Combined, livestock and poultry produce about 1.4 billion tons of manure a year in the United States (1). Most of this manure eventually ends up in the environment, applied either directly as waste or as fertilizer after aerobic digestion or composting (2). This manure contains nutrients, microbes, and unabsorbed antibiotic residue, which have the potential to impact the dynamics of the soil microbial community and contribute to the spread of antimicrobial resistance genes (3). These microbes, originally adapted to the anaerobic condition in the gut, encounter a different environment after excretion (4). Environmental factors such as temperature, pH, moisture content, and oxygen can influence the assembly of the manure microbial community (5). Temperature (6) and pH (7) have been shown to alter the dynamics of manure microbial communities, but little is known about the effect of oxygen on microbial communities.

Oxygen is known as an important modulator of individual bacterial growth (8). Some bacteria require oxygen as a terminal electron acceptor for their growth (aerobes), while others only thrive in the absence of oxygen (anaerobes). The impact of oxygen on the growth and survival of bacteria varies between species (9). Studies on the microbial communities of coastal ecosystems have shown that oxygen modulates the microbiome community composition in a distinct way (10, 11). However, the change in structure of the microbiome in response to oxygen varies depending on the characteristics of the community (12). For example, a community with high diversity was resilient to perturbations caused by changes in oxygen concentration. However, the impact of oxygen on an entire community, competition between microbial populations, fate of populations that are less oxygen tolerant, and especially how oxygen exposure would select for taxa that could be pathogenic or carry resistance genes in the gut is still unclear. Different oxygen gradients have been found in the gut (13), and studies on rats have shown that these different gradients alter the microbiome composition. This supports the idea that aerobic conditions affect the microbiota in the gut and fecal (14–16).

While few studies have studied the impact of oxygen on feces directly, many studies have been completed on composting animal manure or the microbial community dynamics in poultry litter. The litter microbiome of broiler chickens, which is always in an aerobic environment, changes over time. For the first five weeks, there is a decrease in alpha diversity, but then diversity increases afterward (17). The dynamics of the litter microbiome appeared to be associated with the persistence of pathogens like *Campylobacter* in the broiler production system. This dynamic of the broiler litter microbiome is opposite the *in vivo* cecal microbiome development in broilers and turkeys, which has been shown to increase with age (18, 19). Wan et al. (20) reported a similar reduction in diversity and change in the composition of cattle, swine, and chicken manure during composting. This change in microbial structure was host-animal-specific, with an increase in Actinobacteria abundance in manure from all species, a decrease in Chloroflexi abundance in cattle, and an increase in Firmicutes abundance in both pigs and chickens. A similar decrease in diversity of the microbial community during the composting of dairy manure has been previously reported (21, 22), where the abundance of Actinobacteria and Proteobacteria increased while the abundance of Firmicutes was reduced. Microaerobic conditions were also found to affect the succession dynamics of the bacterial community in the solid fraction of the dairy manure storage tank (23).

While compost and litter contain feces and are exposed to oxygen, these studies do not isolate the effect of oxygen on fecal microbial communities. Compost is manure mixed with other organic substances like straw as carbon sources under high temperatures, while litter also has organic carbon over a long period of time. There is a need to isolate the effect of aerobic exposure on the fecal microbiota composition to understand the important transition period from anaerobic to aerobic environment and the complex community processes occurring in these communities. Understanding how aerobic exposure affects the fecal microbiome of each animal will help determine if sampling timing, sample storage, or sample preparation methods should be animal-specific when planning microbiome studies. It will also be valuable in making pen sanitation management decisions because, while animals are housed in pens, they are constantly exposed to aged feces, which could affect the health of both animals and animal handlers. We hypothesized that fecal microbiota communities from different host animals would have host-independent and host-specific responses to oxygen because of the differences in initial microbial communities and chemical properties of each fecal type. We selected broiler chicken, pig, and dairy calf feces because these species represent a wide range of agricultural animals with differing digestive physiologies and diets. We hypothesized further that these community shifts would result in a proliferation of aerobic or facultative anaerobic microbes and potential pathogens. Using 16S rRNA gene amplicon sequencing, we evaluated the dynamic change in the microbiota of calf, piglet, and broiler chick feces when exposed to an aerobic environment in a microcosm experiment over a 21-day period.

## RESULTS

### Succession in the fecal microbiota of broiler chicks

**Fig 1:**
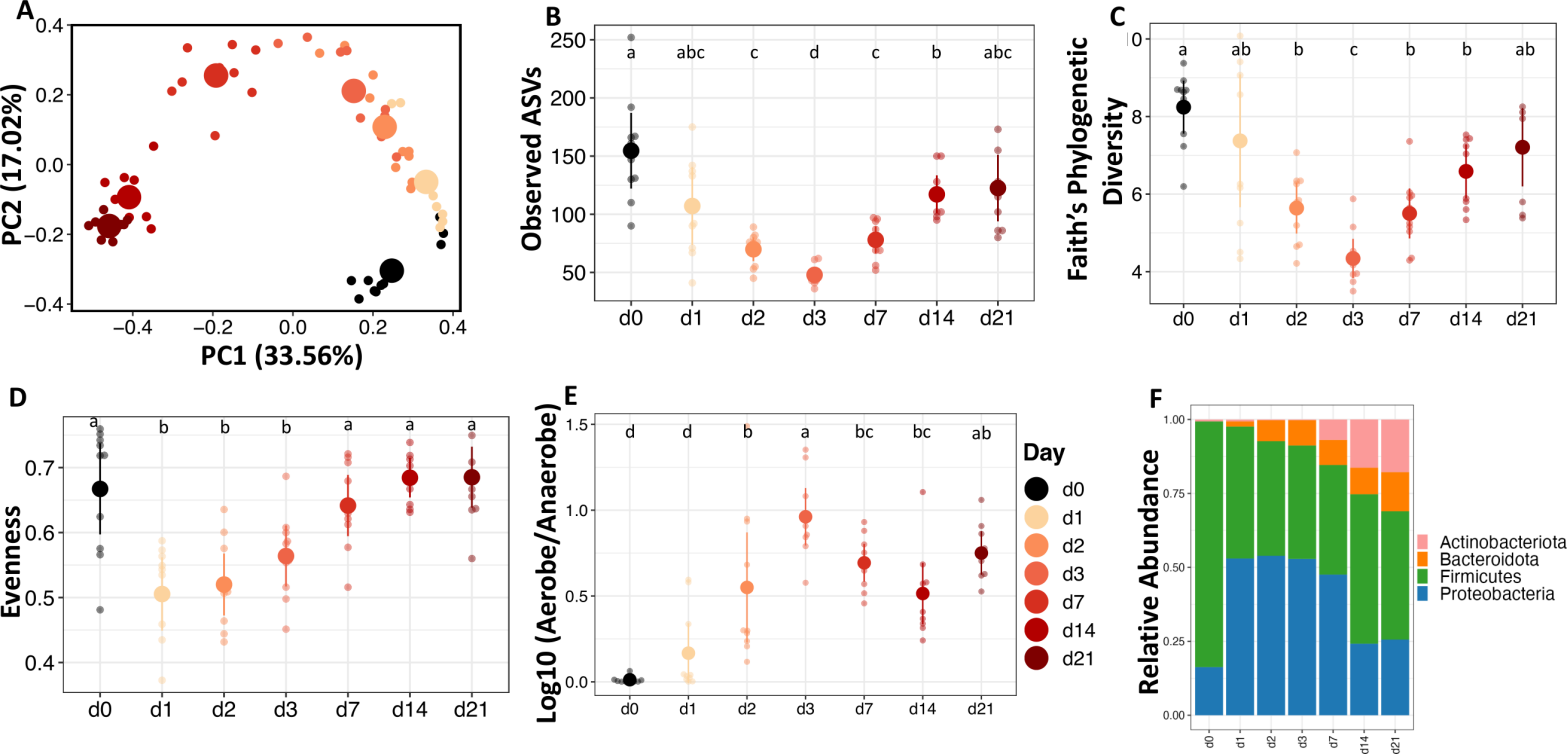
The dynamics of the fecal microbiota of broilers after aerobic exposure are represented by alpha and beta diversity and taxonomy. (**A**) Principal coordinate analysis (PCoA) of Bray-Curtis dissimilarity (beta diversity). Small data points indicate each sample, while large data points indicate the centroid. Alpha diversity measures (**B**) Observed ASVs as a measure of the richness of the community; (**C**) phylogenetic diversity measured by Faith; and (**D**) evenness measured by Pielou. Predicted relative abundance of (**E**) Log ratio relative abundance of predicted Aerobes and Anaerobes by BugBase. (**F**) Taxa bar plot at phylum level showing the average relative abundance of each taxon.

In this study, we expected aerobic exposure to influence bacterial survival and enrichment in fecal communities which would be reflected in alpha and beta diversity metrics. In broiler chick feces, alpha diversity metrics measuring richness (Observed ASVs: number of ASVs), evenness (Pielou’s evenness: difference of relative abundance between ASVs), and phylogenetic diversity (Faith’s PD: breadth of taxonomic diversity) all showed significant changes through time. Observed ASVs (*F* = 26.794, *P <* 0.0001; Fig. 1B), Faith’s phylogenetic diversity, (F = 25.534, *P <* 0.0001; Fig. 1C), and Pielou’s evenness (*F* = 15.936, *P <* 0.0001; Fig. 1D), all decreased by about 50% in the first 3 days following aerobic exposure. However, all these metrics increased from days 7 to 21, returning to day 0 levels. In other words, with aerobic exposure, the fecal community was quickly reduced in the number and phylogenetic breadth of species, while becoming more dominated by fewer species, but after 3 days, all these trends reversed back to a diversity similar to the original diversity. From the diversity analysis, it is not known if this reversal in diversity was due to a return of the same community members or different community members.

Similar to shifts in alpha diversity, an aerobic environment dramatically altered the beta diversity of broiler fecal microbial communities. Beta diversity is measure of community dissimilarity between two samples. Dissimilarity metrics can be based on quantitative community composition (Bray-Curtis), or from qualitative or quantitative differences in phylogenetic relationships between all community members (unweighted or weighted UniFrac, respectively). Interestingly all three beta diversity metrics, Bray-Curtis dissimilarity (*F* = 13.171, *P <* 0.001; Fig. 1A), unweighted UniFrac (*F* = 7.451, *P <* 0.001; Fig. S1a), and weighted UniFrac (*F* = 13.300, *P <* 0.001; Fig. S1b), showed clear clustering on principal coordinates analysis (PCoA) plots based on the day of exposure to an aerobic environment. There was a shift in the community based on days of exposure along PC1, and then the directionality of the community shift was altered after day 3. Samples collected 1 day after aerobic exposure clustered separately from the initial fecal samples (*P <* 0.01); day 2–3 samples clustered together (*P =* 0.413), while day 14–21 samples clustered together (*P =* 0.383). These results indicate that there were qualitative and quantitative shifts in phylogenetically distinct community members and that the ending community was distinct from the original community.

Taxonomic composition analysis showed that the broiler fecal microbiota was initially dominated by members of the phylum Firmicutes, which decreased with aerobic exposure while Proteobacteria, Actinobacteria, and Bacteroidota increased in abundance over time (Fig. 1F). Families *Lachnospiraceae*, *Lactobacillaceae*, and *Ruminococcaceae* decreased rapidly with aerobic exposure (Fig. S1c), while genera *Escherichia/Shigella* and *Acinetobacter* increased substantially beginning on day 1 (Fig. S1d). Interestingly, *Escherichia/Shigella* reduced in abundance after day 3, but *Acinetobacter* maintained a relative abundance of about 10% until day 21. To understand the effect of aerobic exposure on the community functional profile, we predicted the functional potential of the community with PICRUST2. PCoA plots showed that aerobic exposure shifted the predicted community functional profile along PC1 (*F* = 15.770, *P <* 0.001; Fig. S2f). The relative abundance of biofilm formers (*F* = 40.537, *P <* 0.0001; Fig. S2a) and bacteria that likely contain mobile elements (*F* = 6.558, *P <* 0.01; Fig. S2b) increased rapidly after 1 day of aerobic exposure, while that of facultative anaerobes (*F* = 17.372, *P <* 0.0001; Fig. S2c) and gram negatives (*F* = 19.39, *P <* 0.0001; Fig. S2d) increased between days 1–7 but decreased on day 14. On the other hand, the relative abundance of gram positives (*F* = 19.377, *P <* 0.0001; Fig. S2e) decreased between days 1 and 7 but increased on day 14.

**Fig 2:**
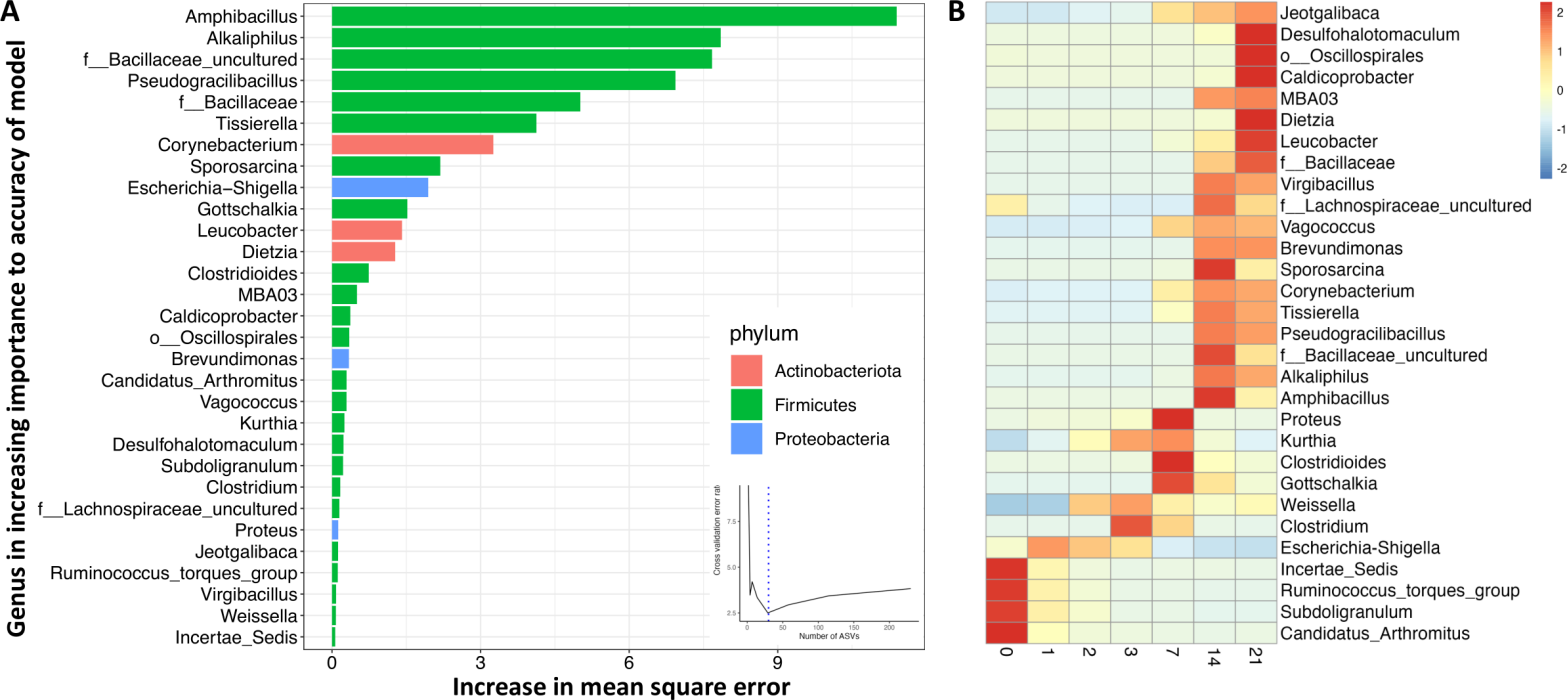
Random forest regression prediction of time of aerobic exposure in broiler fecal samples. (**A**) The 30 most discriminant ASVs identified by random forest regression for predicting time of aerobic exposure in broiler fecal microbiota ranked in order of their importance to the model. The cross-validation error used to select the optimal number of ASVs is inserted. (**B**) Heat map indicating the abundance of these 30 ASVs at each timepoint.

We used a random forest regression machine-learning algorithm to identify a microbiota signature in the broiler chick’s feces that correlates with aerobic exposure by regressing relative abundance at the genus level against the day of exposure. The cross-validation error curve was lowest when 30 ASVs were used to construct the model, which we then used to construct the community succession model (Fig. 2A). The model explained 96.7% of the variation in fecal microbiota in relation to exposure day (Fig. S7a). When considering the abundance of the ASVs at each timepoint (Fig. 2B), 4 of the 30 ASVs were obligate anaerobes and were high in abundance in the initial fecal sample; these 4 ASVs decreased in abundance in a time-dependent manner till day 21. A signature of days 1, 2, and 3 was increased *Escherichia/Shigella* abundance. Various other genera were increased each day during the experiment. As predicted by PICRUSt2 and BugBase, a majority of the genera that increased in abundance during aerobic exposure were aerobes (*Jeotgalibaca*, *Dietzia*, *Leucobacter*, *Vagococcus*, *Bacillaceae*, *Brevudimonas*, *Sporosarcina*, *Corynbacterium*, *Proteus*, and *Kurtia*) or facultative anaerobes (*Amphibacillus* and *Escherichia/Shigella*). Surprisingly, there was an increase in the abundance of some anaerobic bacteria (Desulfohalotomaculum, Oscillospirales, Lachnospiraceae, Tissierella, Alkaliphilus, and Clostridioides) even after 2–3 weeks of exposure to oxygen (Fig. 2B). Interestingly, the log ratio of aerobe to anaerobe quickly rose to nearly 1 on day 3 and then fluctuated afterward (Fig. 1E), which corresponds with an increase in anaerobes after 2 weeks.

### Succession in the fecal microbiota of calves

**Fig 3:**
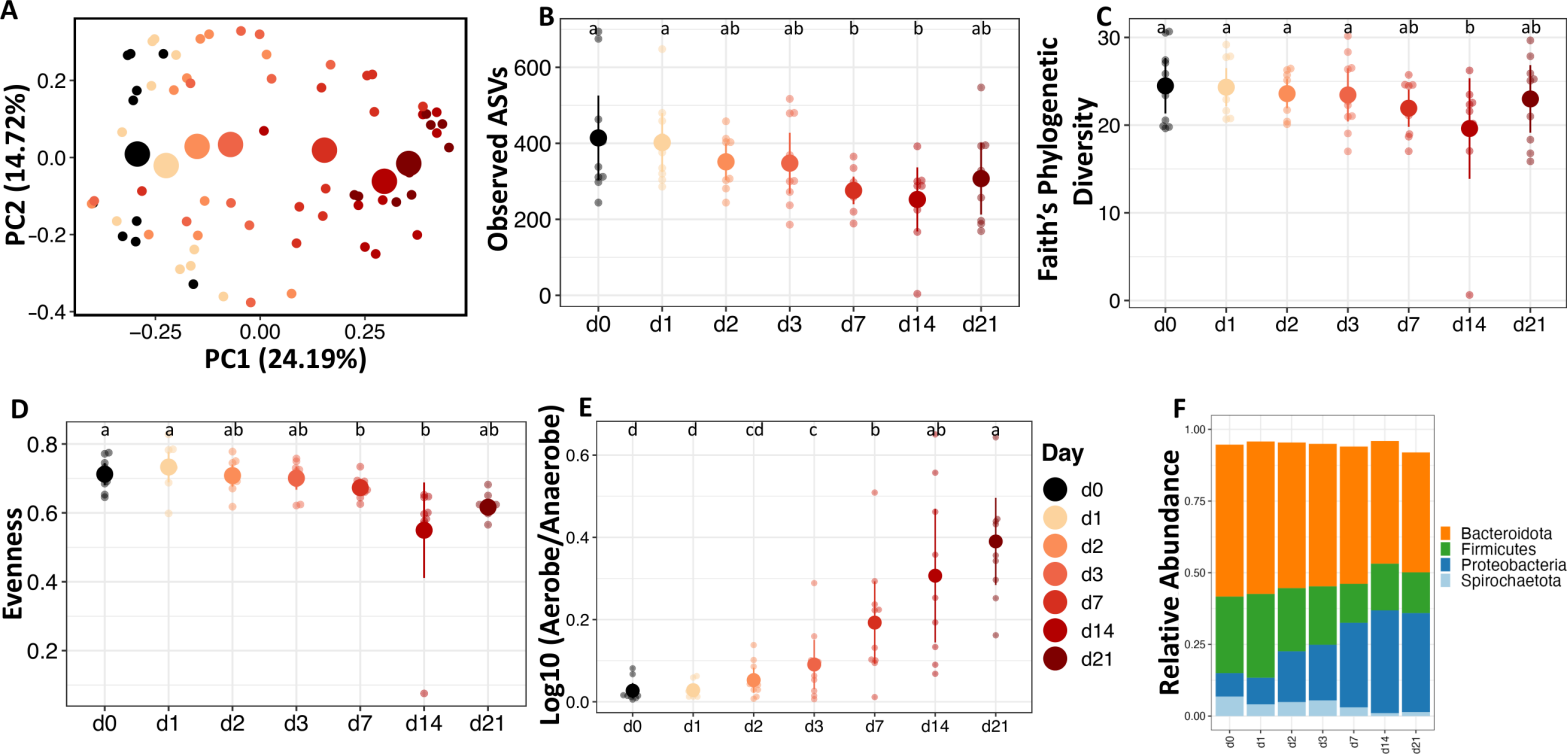
The dynamics of the fecal microbiota of calves after aerobic exposure are represented by alpha and beta diversity and taxonomy. (**A**) PCoA of Bray-Curtis dissimilarity (beta diversity). Small data points indicate each sample, while large data points indicate the centroid. Alpha diversity measures (**B**) Observed ASVs as a measure of the richness of the community; (**C**) phylogenetic diversity measured by Faith; and (**D**) evenness measured by Pielou. Predicted relative abundance of (**E**) Log ratio relative abundance of predicted Aerobes and Anaerobes by BugBase. (**F**) Taxa bar plot at phylum level showing the average relative abundance of each taxon.

Similar to what was described previously, we used multiple diversity metrics to capture the abundance and distribution of taxa in calf feces microcosms. The microbiota community richness, evenness, and phylogenetic diversity in calf feces was initially somewhat resilient to aerobic exposure. The number of observed ASVs (*F* = 4.003, *P <* 0.05; Fig. 3B), Faith’s phylogenetic diversity (*F* = 2.460, *P <* 0.05; Fig. 3C), and Pielou’s evenness (*F* = 13.688, *P <* 0.0001; Fig. 3D) remained the same from day 1 through day 3, but decreased on days 7 and 14, followed by a slight increase on day 21 compared to day 14.

Comparison of community structure by PERMANOVA between days indicated a similar pattern as alpha diversity. Bray-Curtis dissimilarity (*F* = 3.455, *P <* 0.001; Fig. 3A), unweighted UniFrac (*F* = 2.105, *P <* 0.001; Fig. S3a), and weighted UniFrac (F = 4.912, *P <* 0.001; Fig. S3b). The communities exhibited a linear progression over time, but days 0–3 clustered together separately from days 7 to 21. In general, compared to the chicken samples, there was a delay in the change in structure of the calf microbiota community after exposure to an aerobic environment.

Taxonomic composition analysis showed that after 7 days, at the phylum level, Proteobacteria increased while Spirochaetes decreased (Fig. 3F). At the genus level, *Prevotella*, *Succinivibrio*, and *Treponema* decreased, while *Acinetobacter*, *Bacteroides*, *Escherichia/Shigella*, *Flavobacterium*, and *Strenotrophomonas* increased in abundance (Fig. S3d). The beta diversity comparison of the predicted functional potential of the community shifted along PC1 with time, with days 0 and 1 clustering together, days 2 and 3 clustering together, while days 7 and 21 clustered together (*F* = 8.678, *P <* 0.001; Fig. S4f). Phenotype prediction from BugBase showed that the log ratio of aerobe/anaerobe (*F* = 11.53, *P <* 0.00001; Fig. 3E) increased after day 2, while there was no difference between facultative anaerobes (Fig. S4c) or those that contain mobile elements (Fig. S4b). The relative abundance of biofilm formers (*F* = 16.549, *P <* 0.0001; Fig. S4a) and gram negatives (*F* = 7.195, *P <* 0.01; Fig. S4d) increased after day 3; gram positives (*F* = 5.929, *P <* 0.01; Fig. S4e) decreased on day 7.

**Fig 4:**
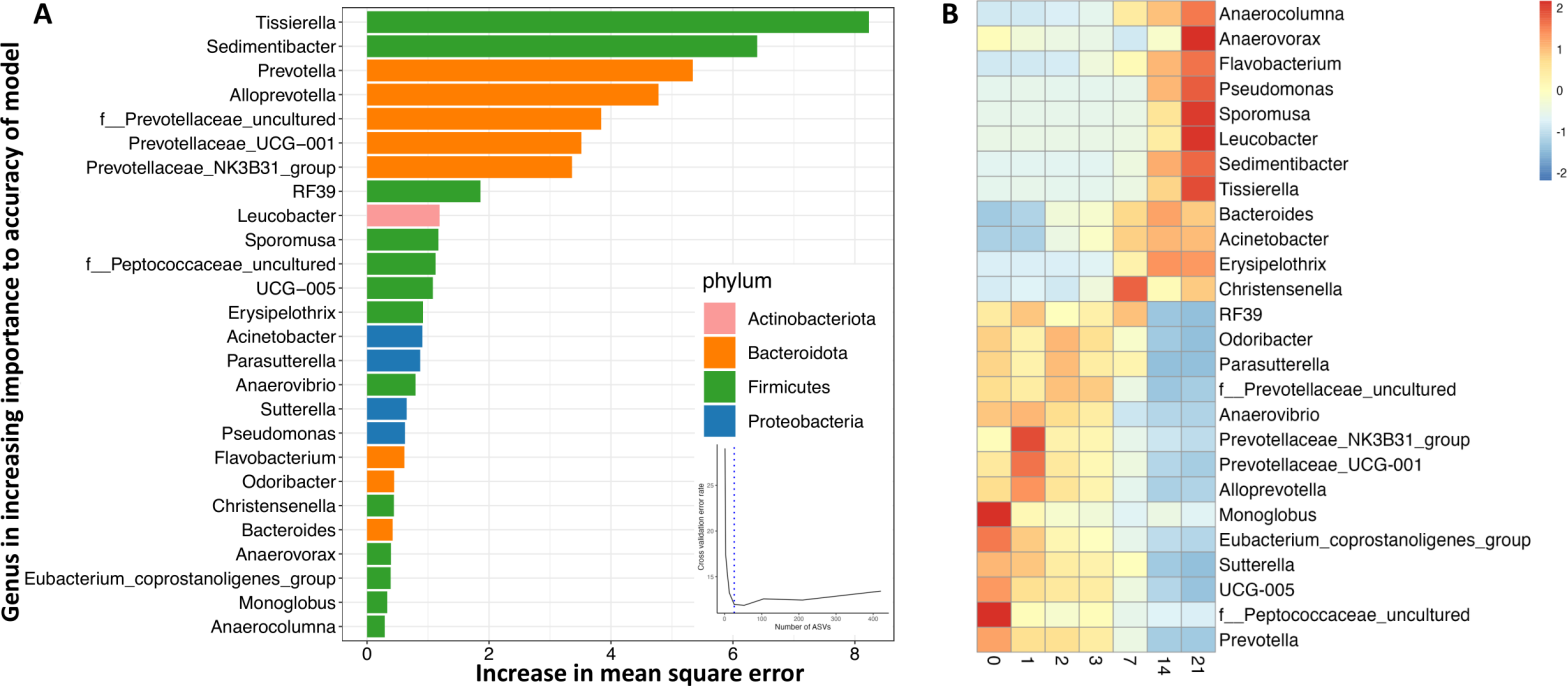
Random forest regression prediction of time of aerobic exposure in calf fecal samples. (**A**) The 26 most discriminant ASVs identified by random forest regression for predicting time of aerobic exposure in calf fecal microbiota ranked in order of their importance to the model. The cross-validation error used to select the optimal number of ASVs is inserted. (**B**) Heat map indicating the abundance of these 26 ASVs at each timepoint.

A random forest regression machine-learning algorithm was used to identify microbiota signatures in calf feces that correlates with aerobic exposure. The model was built by regressing the relative abundance of genus-level taxa against the day of exposure. The model explained 80.8% of the variation in fecal microbiota based on exposure day (Fig. S7b). The cross-validation error curve was lowest with 26 ASVs used in the predictive model (Fig. 4A). The predictive ASVs display clear time-dependent abundance signatures (Fig. 4B). Early aerobic exposure was characterized by a decrease in abundance of anaerobic Prevotella-like ASVs (*Prevotella*, *Alloprevotella*, Prevotellaceae_UCG-001, and Prevotellaceae_NK3B31) between days 0 and 7, while longer-term aerobic exposure was characterized by an increase in abundance of *Tissierella*, *Sedimentibacter*, *Leucobacter*, *Sporomusa*, *Anaerovorax,* and *Acinetobacter* between days 7 and 21. All ASVs that decreased were anaerobic; although five anaerobic ASVs increased (*Anaerocolumna*, *Anaerovorax*, *Sporomusa*, and *Bacteroides*), another six increased ASVs were either facultative anaerobes (*Sedimentibacter* and *Erysipelothrix*) or aerobes (*Flavobacterium*, *Pseudomonas*, *Leucobacter*, and *Acinetobacter*), which indicates that the most distinctive shifts in the microbial community were toward an increase in aerobic or facultative aerobic organisms (Fig. 4B).

### Succession in the fecal microbiota of piglets

**Fig 5:**
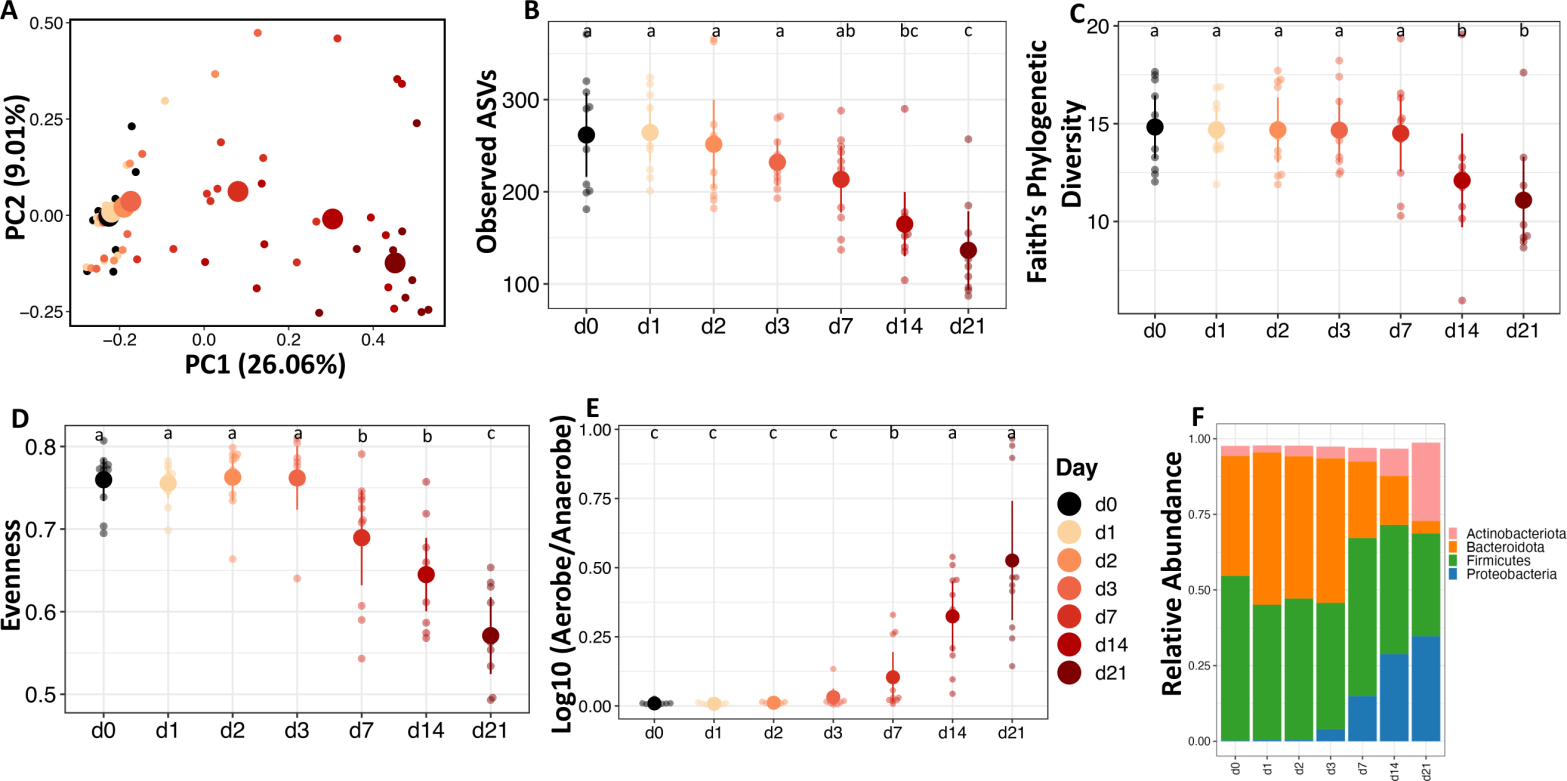
The dynamics of the fecal microbiota of piglets after aerobic exposure are represented by alpha and beta diversity and taxonomy. (**A**) PCoA of Bray-Curtis dissimilarity (beta diversity). Small data points indicate each sample, while large data points indicate the centroid. Alpha diversity measures (**B**) Observed ASVs as a measure of the richness of the community; (**C**)phylogenetic diversity measured by Faith; and (**D**) evenness measured by Pielou. Predicted relative abundance of (**E**) Log ratio relative abundance of predicted aerobes and anaerobes by BugBase. (**F**) Taxa bar plot at phylum level showing the average relative abundance of each taxon.

Similar to the calf fecal community, richness, evenness, and phylogenetic diversity all eventually decreased in piglet feces. The number of Observed ASVs (F = 11.122, *P <* 0.0001; Fig. 5B), Faith’s phylogenetic diversity (*F* = 6.813, *P <* 0.0001; Fig. 5C), and Pielou’s evenness (*F* = 18.602, *P <* 0.0001; Fig. 5D) initially maintained some level of stability following aerobic exposure but then decreased after day 7 or 14. After the diversity levels began to decline, there was no increase in diversity at later time points.

Comparison of community composition between days indicated a similar pattern as alpha diversity. There was stratification in the data, with days 0–3 clustering together, while samples from days 7, 14, and 21 changed in a time-dependent manner (*P <* 0.05). The Bray-Curtis (*F* = 4.107, *P <* 0.001; Fig. 5A), unweighted UniFrac (*F* = 1.950, *P <* 0.001; Fig. S5a), and weighted UniFrac (*F* = 12.353, *P <* 0.001; Fig. S5b) PCoA plots showed a shift in the clustering pattern along PC1 with aerobic exposure. Similar to the calf fecal community, there was an initial resistance to community shift after exposure to an aerobic environment.

The initial community was primarily made up of Bacteroides and Firmicutes members (Fig. 5F). From day 3 to day 21, the phyla Actinobacteria and Proteobacteria gradually increased to a combined abundance of about 60% of the community. Members of the phylum Bacteroides were most impacted by aerobic exposure, with an initial relative abundance of about 40% and a final relative abundance of about 5% (Fig. 5F). At the family level, *Lachnospiraceae*, *Prevotellaceae,* and *Ruminococcaceae* were most affected, as their combined abundance decreased from 60% to about 3% after 21 days of aerobic exposure (Fig. S5c). At the genus level, the abundance of *Blautia* and *Prevotella* decreased, while that of *Acinetobacter*, *Brevundimonas*, *Corynbacterium*, *Enterococcus*, and *Tissierella* increased with aerobic exposure (Fig. S5d). Early initial stability of the piglet fecal microbiota in an aerobic environment was also observed in the predicted relative abundance of the different bacterial phenotypes and community functional profile. The predicted functional profile on days 0–3 clustered together, while days 7–21 changed in a time-dependent manner (*F* = 9.493, *P <* 0.001; Fig. S6f). The log ratio of aerobe/anaerobe (*F* = 22.88, *P <* 0.0001; Fig. 5E), relative abundance of biofilm formers (*F* = 22.033, *P <* 0.0001; Fig. S6a), and those that contain mobile elements (*F* = 7.200, *P <* 0.0001; Fig. S6b) all increased beginning on day 7, while that of facultative anaerobes (*F* = 18.110, *P <* 0.001; Fig. S6c) increased on day 14. There was no difference in the relative abundance of gram negative (*F* = 1.426, *P >* 0.05; Fig. S6d) and gram positive (*F* = 2.650, *P >* 0.05; Fig. S6e).

**Fig 6:**
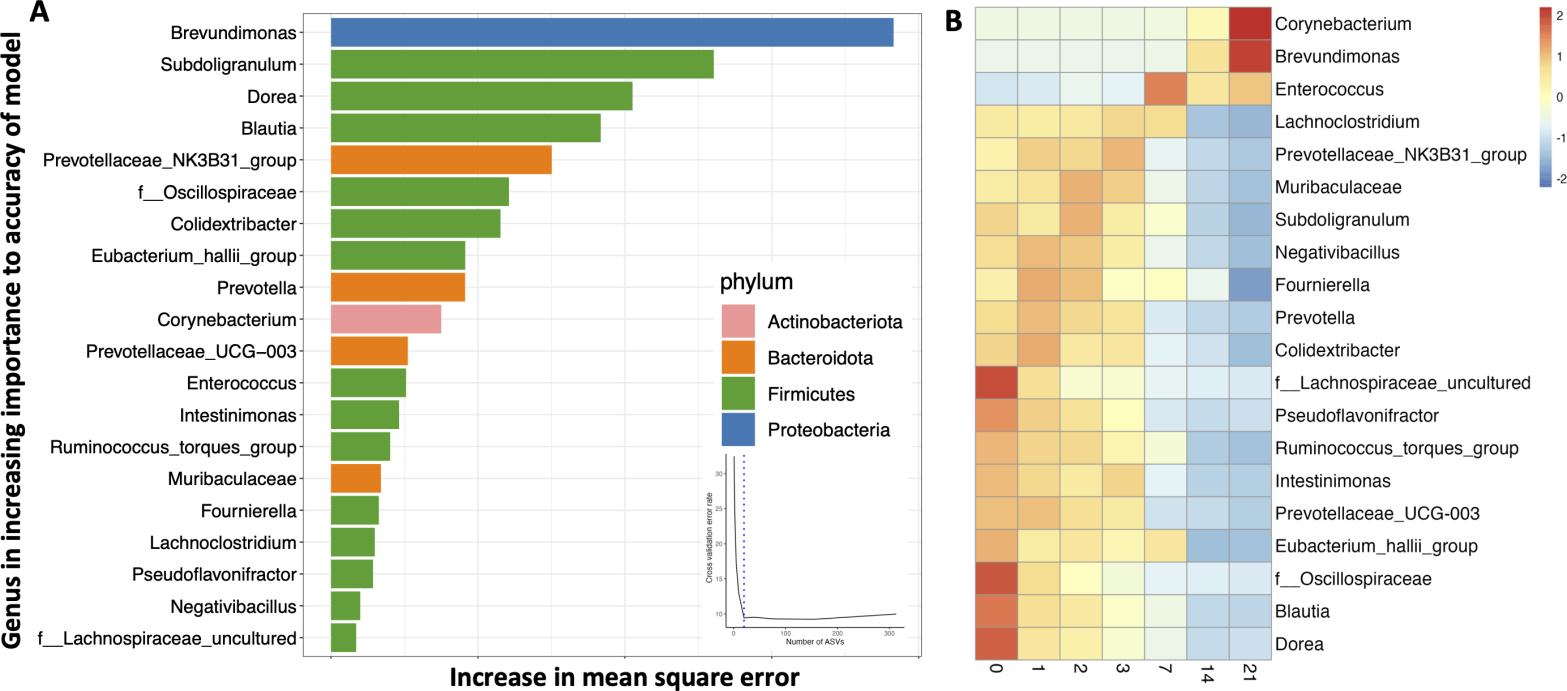
Random forest regression prediction of time of aerobic exposure in piglet fecal samples. (**A**) The 20 most discriminant ASVs identified by random forest regression for predicting time of aerobic exposure in piglet fecal microbiota ranked in order of their importance to the model. The cross-validation error used to select the optimal number of ASVs is inserted. (**B**) Heat map indicating the abundance of these 20 ASVs at each timepoint.

A random forest machine-learning algorithm identified 20 microbial genera as signatures of aerobic exposure (Fig. 6A). The model explained 85.6% of the variation (Fig. S7c). *Dorea*, *Blautia,* and an *Oscillospiraceae* unclassified ASV, which are anaerobes, all decreased immediately after exposure. On the other hand, *Enterococcus*, a facultative anaerobe, *Brevudimona*s, and *Corynbacterium*, which are both aerobic, all increased between days 7 and 21 (Fig. 6B).

### Animal fecal-microbiota-specific responses to aerobic exposure

**Fig 7:**
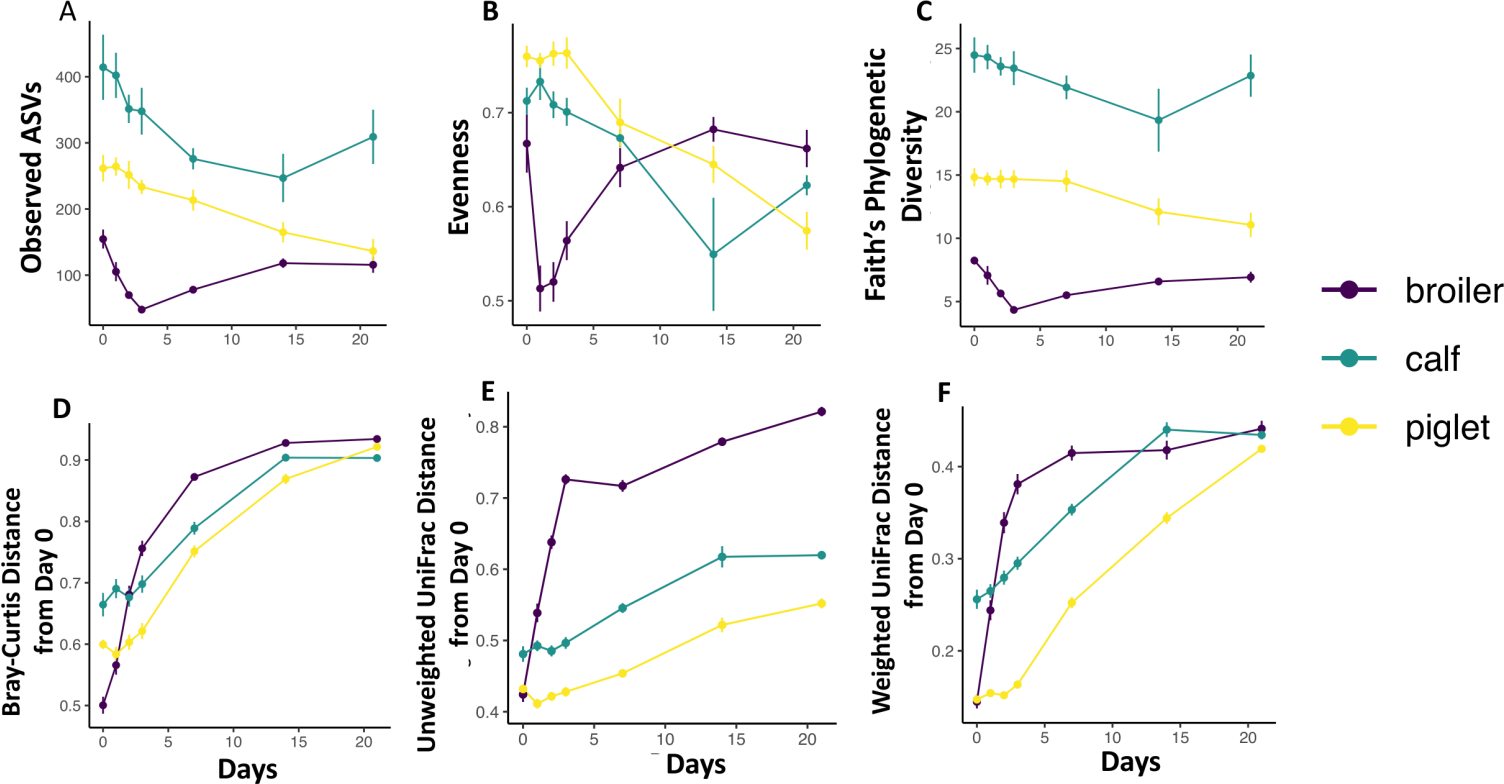
Changes in alpha and beta diversity of the fecal microbiota of broilers, calves, and piglets after aerobic exposure. Change in (**A**) number of observed ASVs, (**B**) evenness, (**C**) Faith’s phylogenetic diversity, and (**D**) Bray-Curtis dissimilarity distance of each sample from day 0; (**E**) unweighted UniFrac distance of each sample from day 0; and (**F**) weighted UniFrac distance of each sample from day 0.

**Fig 8:**
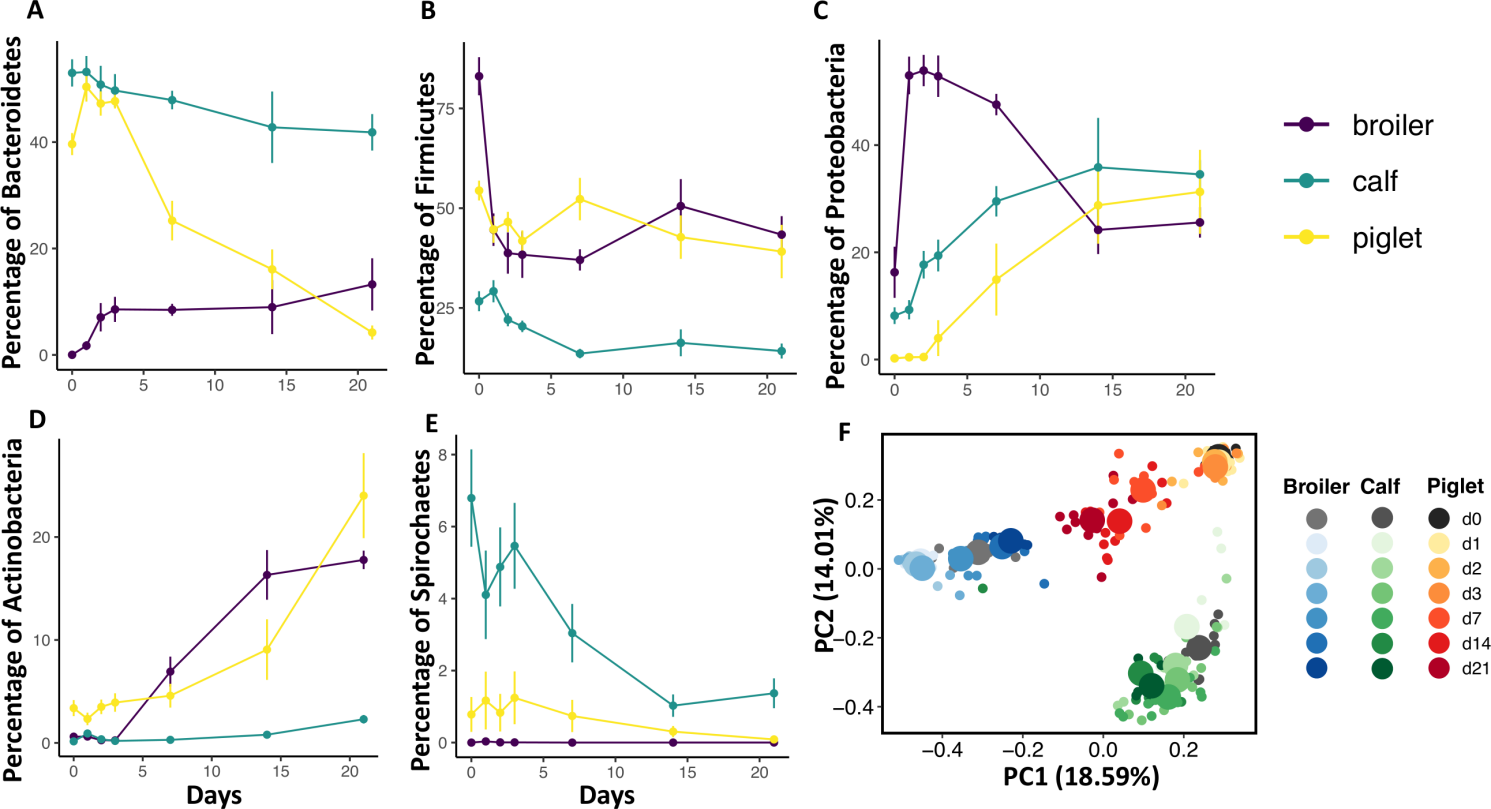
Changes in proportion of the five dominant phylum of the fecal microbiota of broilers, calves, and piglets after aerobic exposure. Proportion of (**A**) Bacteroidetes, (**B**) Firmicutes, (**C**) Proteobacteria, (**D**) Actinobacteria, and (**E**) Spirochaetes. PCoA of Bray-Curtis dissimilarity (beta diversity) of all three species combined, small data points indicate each sample while large data point indicate the centroid.

Alpha diversity metrics (observed ASV, evenness, and Faith’s PD) decreased in all animal fecal communities after aerobic exposure, but this change was rapid (days 1–3) in broiler samples (Fig. 7A through C), while both calf and piglet samples showed some degree of initial stability until days 7–14 before decreasing more sharply. Broiler diversity began to rebound when the calf and piglet diversity initially began to decrease. Similar to alpha diversity, beta diversity metrics (Bray-Curtis, unweighted UniFrac, and weighted UniFrac) changed rapidly in broiler samples between days 1 and 3 (Fig. 7D through F), and the change subsequently became gradual until day 21. In both calf and piglet samples, there was also initial stability in beta diversity, followed by a larger shift in the community after day 3. When comparing the community succession from all three animals, Bray-Curtis PCoA plot showed that all three initial animal communities were clearly distinct, and broilers and piglets converged in similar direction while calf samples transitioned in a different trajectory (Fig. 8F).

The proportion of Bacteroidetes was high initially in both calf and piglet samples (Fig. 8A), but it decreased rapidly in the piglet samples and less rapidly in the calf samples, while the proportion of Bacteroidetes was low in broiler samples but increased over time of aerobic exposure (Fig. 8A). The proportion of Firmicutes in broiler and calf samples decreased immediately after aerobic exposure and remained lower until day 21 (Fig. 8B); the decrease was more rapid in broiler samples. The proportion of Actinobacteria and Proteobacteria increased rapidly after aerobic exposure. Proteobacteria increased rapidly in the broiler samples but decreased after day 3 (Fig. 8C), while there was a 24 and 48 hour lag time in the calf and piglet samples before their proportions increased (Fig. 8C). In both broiler and piglet samples, there was an initial lag time of 72 hours before Actinobacteria increased after aerobic exposure (Fig. 8D). Spirochaetes were more abundant in the calf samples but decreased rapidly after aerobic exposure (Fig. 8E). Overall, while fecal samples from all three animals were sensitive to aerobic exposure, broiler samples were the most sensitive, and the changes in the fecal microbiome were animal specific.

## DISCUSSION

Despite evidence that aerobic exposure influences the microbiota dynamics, the response of fecal microbiota specifically to aerobic exposure from different agricultural animals is not well known. Here, we explored the effect of aerobic exposure on the fecal microbiota succession dynamics of broilers, calves, and piglets over a 21-day period. We hypothesized that the effect of aerobic exposure on animal feces would vary between animal species because of the variation in the initial communities present and chemical properties of each feces type [e.g., broiler excreta contains both feces and urine and contains more organic matter than feces from both pigs and cattle (24)], and there would be an increase in abundance of aerobes, facultative anaerobes, and potential pathogens.

The impact of aerobic exposure on the fecal microbiota was, in some ways, similar in all animals. The bacterial communities in all feces changed in a time-dependent fashion; alpha diversity and the relative abundance of anaerobic species decreased, but the relative abundance of aerobic species, Proteobacteria, Actinobacteria, biofilm formers, and species predicted to contain mobile elements increased. While a rise in *Escherichia/Shigella* was expected to occur, in reality, it was *Acinetobacter* that increased in relative abundance in the feces of all three animal species. This is interesting because our earlier work has potentially implicated *Acinetobacter* as a carrier of multiple antibiotic resistance genes in swine manure (25), and antibiotic-resistant *Acinetobacter* has been observed to increase in swine farm groundwater (26).

Despite the similarities in succession, animal-specific effects were also observed based on the initial difference in the composition of the microbiota. The broiler fecal microbiota responded the quickest to aerobic exposure, with the diversity changing almost daily. As previously reported (27), Firmicutes dominated the excreta microbiota of broilers, making up about 83% of the initial microbiota but decreasing to 44% after 24 hours of exposure and remaining at 43% on day 21. Firmicutes decreased as expected, but Proteobacteria and Actinobacteria increased from 16% to 25% and 0.6% to 18%, respectively. However, the number of observed ASVs initially decreased before increasing after the third day of aerobic exposure. The decrease in the number of ASVs was due to the reduction in the population of Firmicutes, while the subsequent increase in the number of ASVs after day 3 was due to the increase in the population of Actinobacteria, Bacteroides, and Proteobacteria. *Escherichia/Shigella*, a genus that includes potential opportunistic pathogens (28), increased after initial exposure but decreased in abundance on day 7. Similar increases in the abundance of Proteobacteria and Actinobacteria have been reported in the chicken litter microbiome (29), suggesting the increase in their abundance may be associated with the availability of oxygen.

Among the random forest identified biomarkers, anaerobic bacteria in the initial samples decreased over time while aerobes and facultative anaerobes increased. Some anaerobes also increased after aerobic exposure. Although the samples were mixed daily in efforts to maintain an aerobic condition, this may suggest that pockets of anaerobic environments were still maintained in the samples during aerobic exposure. Random forest predicted that the segmented filamentous bacteria (SFB), *Candidatus* arthromitus, which has been associated with improved T cell response and gut health in humans and different animal species (30), was decreased after aerobic exposure. *Jeotgalibacca*, *Corynbacterium*, and *Wiessella*, which were all increased after aerobic exposure, have also been shown to increase in litter microbiome. This suggests that these predicted taxa could be markers of different lengths of aerobic exposure (29). Unlike the broiler microbiota, the fecal microbiota of calves and piglets, which had a higher diversity, were stable after initial aerobic exposure. Previous studies in coastal sediments have reported resilience by microbial communities with higher diversity during changes in oxygen concentration (12). The diversity eventually decreased, and the abundance of beneficial families like *Lachnospiraceae, Ruminococcaceae,* and *Prevotellaceae* decreased, while the abundance of *Acinetobacter*, *Corynbacterium*, *Tissierella*, *Enterococcus*, and *Enterobacteria*, which can also be found in high abundance in manure (6, 20, 31), increased.

The fecal microbiota of the three animals was affected by aerobic exposure in a distinct way. This might be due to differences in the physiology of the animals, which can affect the physical properties of the voided feces. For example, the transit time of the materials going through the gastrointestinal tract of broilers is shorter, and broiler excreta contains both feces and urine, higher in nutrient density, and lower in viscosity. This will allow for oxygen circulation in the community and nutrient availability for emerging microbes. This physiochemical property of the broiler excreta microbiota may be responsible for the high impact of aerobic exposure on its composition and diversity. This is different from the calf and piglet feces, which are less nutrient dense and more viscous. The physicochemical properties of calf and piglet feces may allow the maintenance of pockets of anaerobic environment.

Another possible explanation for the animal-specific community succession following aerobic exposure is the difference in the initial composition of the microbiota among the three species. Although it is difficult to separate this effect from that of the physiochemical properties, each species appeared to continue to change based on its unique starting composition. The initial proportion of Bacteroidetes in broiler fecal microbiota was low but continued to increase to 16% on day 21, while that of piglets decreased rapidly from 40% to less than 5%. The proportion of Firmicutes in broiler samples decreased by half (from 80% to 40%) on day 1 of sampling, while it remained relatively constant in calves and piglets throughout the exposure period. While there were differences between animals, in general, the proportion of dominant phyla such as Bacteroidetes and Firmicutes decreased in all three species, while Proteobacteria and Actinobacteria, which existed in lower proportions, increased to become dominant.

The results described in this study could have important applications to animal production from a one-health perspective. First, Proteobacteria and Actinobacteria are important in the decomposition of organic substances, but they also contain several important opportunistic pathogens for animals and humans (32, 33). The proliferation of these pathobionts may have significant implications for farm sanitation decisions because they may be found in feces that both humans and animals come in contact with. Understanding when these pathobionts become more abundant during aerobic exposure could help determine sanitation practices and management decisions on farms because of the potential risk of transferring pathogens (34) and antimicrobial resistance genes (35) to soil when manure is land applied.

Manure is applied to agricultural fields to supply the necessary nutrients required for crop growth. However, feces or manure contain not only physiochemical substances but also microbes, which can be transmitted into the soil and alter the dynamics of the soil microbial community (36). Different oxygen gradients also exist in the soil, which can affect the soil microbial community structure (37) when manure is applied to the soil. However, the impact of these aerobic gradients present in the soil on the manure microbiome and soil microbiome when mixed together is unclear. Further research is needed to explore the interaction of the aerobic condition in the soil, soil microbiome, and manure microbiome when mixed.

Understanding how oxygen impacts microbial communities could also have important applications for host-microbiome interactions. The gut microbiome is a complex and dynamic community (38), dominated by predominantly anaerobic bacteria (39), essential for digestion, immune system development (40), and pathogen resistance (41). Because of these factors, maintaining stable gut microbial composition is critical for maintaining gut homeostasis and the health of the host. Various human and animal diseases have been associated with dysbiosis of the microbiota. Similar to what we observed with the aeration of fecal communities, microbiota dysbiosis in the gut generally leads to a decrease in microbial diversity (42), a decrease in Bacteroides and Firmicutes, and an increase in facultative anaerobes like the family Enterobacteriaceae (43–45).

In some cases, we observed that the fecal community responded very quickly to an aerobic environment. Similarly, Taguer et al. (46) reported in an *in vitro* experiment that 6 hours of aerobic exposure impaired bacterial membranes. *In vivo* studies have suggested that perturbations like antibiotic use can increase oxygen concentration in the lumen and cause microbiome dysbiosis, which shifts the composition of the gut microbiome from obligate anaerobes to facultative anaerobes (47, 48). This all suggests that oxygen concentration is important to gut microbiota dynamics. Therefore, it is important to understand factors that lead to increased oxygen content in the gastrointestinal tract and treatments that will decrease oxygen in the gut. The consequence of increased oxygen, leading to an increased abundance of Enterobacteriaceae, may increase the relative abundance of antibiotic-resistant bacteria, potentially horizontal gene transfer, and other survival mechanisms. Third, with the increase in studies to develop microbiota modulatory therapies like fecal microbiota transplant, consideration of aerobic exposure to donor samples could be important in designing experiments.

### Conclusion

This study demonstrated that an aerobic environment significantly altered the diversity and composition of the aged-fecal microbiota from three livestock animals in an animal-specific manner. This could be because of the difference in physiochemical properties of the feces or the difference in the initial composition of the community. The altered community composition appeared to be driven by a decrease in the relative abundance of anaerobic species and an increase in the abundance of aerobic species. Interestingly, it was the increase in *Acinetobacter* that was consistent in the feces from all three animals. The broiler microbiota appeared to be the most sensitive to aerobic exposure, with a daily change in microbiota composition and a drastic decrease in diversity, while the aged-fecal microbiota of both piglet and calf, which had higher initial bacterial diversity, were somewhat resilient to aerobic exposure for about 3 days. These results could improve farm sanitation decisions and the design of microbiota studies for different livestock species.

## MATERIALS AND METHODS

### Sample collection, DNA extraction, and library preparation

Pig and calf samples were collected according to protocols 2108002177 and 1808001783, respectively, approved by the Purdue University Institutional Animal Care and Use Committee (IACUC). No IACUC approval was required for broiler excreta collection since the animals were not handled. Fresh fecal samples were collected from 10 cages of broiler chicks (approximately 9 days old), 10 calves (approximately 56 days old), and 10 piglets (approximately 28 days old) at the Animal Science Research and Education Center (ASREC) at Purdue University. Fecal samples from calves and piglets were collected by rectal palpation, and the samples were immediately placed on ice. For broilers, pans lined with paper were placed under each cage, and excreta samples were collected within 30 minutes. The fecal samples were transported on ice from the farm to the laboratory, where they were placed in glass bottles in the fume hood.

Fresh samples were collected immediately as a baseline (day 0) after mixing the samples. The bottles were then left open for air to circulate through the samples, and the samples were stirred daily to allow air penetration. Samples were then collected on days 1, 2, 3, 7, 14, and 21 after mixing to represent different times of exposure to the aerobic condition. Genomic DNA was extracted from the aged-fecal samples collected on the different days with the PowerFecal Kit (Qiagen, Germantown, MD, USA). A 16S rRNA library was prepared according to the protocol described by Kozich et al. (49). The V4 region of the 16S rRNA gene was amplified by PCR. The PCR amplicons were purified, and their concentration was normalized with the SequalPrep Normalization Plate kit (Thermo Fisher Scientific, Frederick, MD, USA). The amplicons were then pooled and sequenced with the Illumina MiSeq sequencer (2 × 250 paired ends) at the Purdue Genomic Core Facility.

### Sequence processing

Raw sequences obtained from the 16S rRNA sequencing were analyzed with Quantitative Insight into Microbial Ecology (QIIME2 v. 2021.11) (50). Low-quality reads were removed with DADA2 (51). Both forward and reverse sequences were trimmed at position 13, while they were truncated at position 250 to allow a 50th percentile quality score >37. At denoising with DADA2, a total of 3,256,065 sequences for broilers, 7,162,767 sequences for calves, and 3,535,051 sequences for piglets were retained. Forward and reverse reads were then merged and rarefied at 12,422 reads, 21,944 reads, and 19,260 reads per sample for broilers, calves, and piglets, respectively, before calculating diversity metrics. At rarefaction, four samples (one each from days 1 and 14, and two from day 21) were lost in broilers, while two samples (one each from days 14 and 21) and one sample (day 3) were lost in calf and piglet, respectively. To obtain Bray-Curtis dissimilarity of the three species combines, all samples were reprocessed and rarefied at 12,422 reads. Taxonomy was then assigned by a classifier trained with the V4 region (515F/806R) of the Silva database (version 138) (52–54).

Observed ASVs as a measure of richness (55), Pielou’s index as a measure of evenness (56), and Faith’s index as a measure of phylogenetic diversity (57) were used to estimate alpha diversity. Bray-Curtis dissimilarity distance (58), unweighted UniFrac distance (59), and weighted UniFrac distance (60) were used to estimate beta diversity as a measure of dissimilarity in microbial community structure between samples. All beta diversity metrics were plotted as PCoA plots in R (v 4.2.2) (61). The functional potential of the community was predicted with Phylogenetic Investigation of Communities by Reconstruction of Unobserved States 2 (PICRUST2) (62) using the Evolutionary Placement Algorithm (EPA-ng) (63), GAPPA (64), and CASTOR (65). We then used BugBase (66) to predict microbial phenotypes present in the community using the Kyoto Encyclopedia of Genes and Genomes (KEGG) (67), Pathosystems Resource Integration Center (PATRIC) (68), and Integrated Microbial Genomes (IMG) (69). The structure of the community functional profile was plotted as principal coordinate analysis (PCoA), while the predicted phenotypes were presented as relative abundance.

### Statistical analysis

Alpha diversity metrics (Observed ASVs, Pielou Evenness, and Faith’s phylogenetic diversity) and the predicted microbial phenotypes were analyzed with General Linear Mixed Model with the animal (for both calf and piglet) and cage (for broiler) used as random factor while Day was considered a fixed factor. The Kenward-Roger approximation was used for the approximation of degree of freedom, and *P*-values were adjusted for multiple comparisons using Holm’s correction. The random factor was computed with the afex package (70) to include both random intercept and random slope without their correlation for both broiler and calf, except for Faith’s phylogenetic diversity in calf where the model was written to only include a random slope because the model did not converge. For piglets, the random factor in the model was written to only include random slope because the model did not converge. Normality of residuals and homogeneity of variance were checked, and dependent variables that did not meet the assumption were either log transformed, or square root transformed. Permutational multivariate analysis of variance test (PERMANOVA, 999 permutations) (71) was used to test the statistical difference in distance between samples with Beta diversity metrics (Bray-Curtis dissimilarity index, unweighted UniFrac distance, weighted UniFrac distance, and the structure of the functional potential) across days in all three species, and PCoA was used for illustration. To calculate the proportion of the five dominant phyla in all three species, the sequence count of each phylum was divided by the number of sequences at which each species was rarefied.

Random forest regression analysis was performed with the RandomForest package in R (v.4.7-1.1) (72) to identify the taxa that contributed the most to the prediction model. The rarified ASV tables collapsed at the genus level were used for random forest regression analysis. The samples (*n* = 70) were divided into a training set [80% of samples (*n* = 53 for broiler, *n* = 55 for calf, and *n* = 55 for piglet)] and a testing set [20% of samples (*n* = 13 for broiler, *n* = 13 for calf, and *n* = 14 for piglet)]. All plots were made with the ggplot2 package (73), and all statistical analyses were performed in R (v 4.2.2) except for PERMANOVA, which was done in QIIME2.

## Data Availability

All raw sequencing reads are available in the NCBI sequence read archive (SRA) under the project accession PRJNA1020266. All additional files and scripts used in data analysis for this study are available at https://github.com/oluwapaul/Fecal-Succession.

## SUPPLEMENTAL MATERIAL

Supplemental figures spectrum.04084-23-s0001.pdf Fig. S1-S7.

ASM does not own the copyrights to Supplemental Material that may be linked to, or accessed through, an article. The authors have granted ASM a non-exclusive, world-wide license to publish the Supplemental Material files. Please contact the corresponding author directly for reuse.
