# Supplementary material for: Temporal dynamics of fecal microbiota community succession in broiler chickens, calves, and piglets under aerobic exposure
Source: Microbiol Spectr. 2024 May 8;12(6):e04084-23. doi: 10.1128/spectrum.04084-23 (PMC11237419; doi:10.1128/spectrum.04084-23)
Supplement: Supplemental figures — Fig. S1-S7. [file spectrum.04084-23-s0001.pdf]

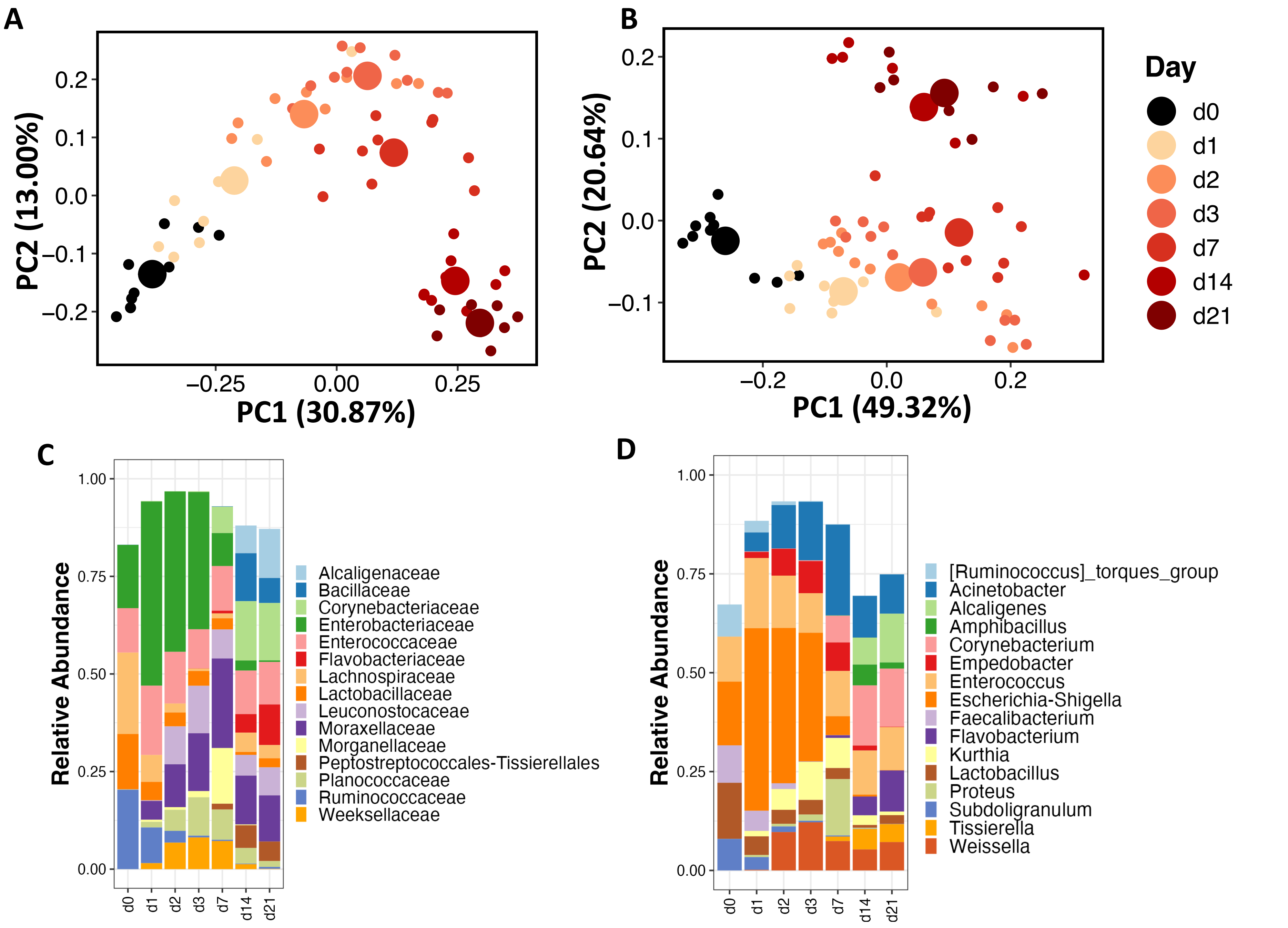
A
PC2 (13.00%)
PC1 (30.87%)
B
PC2 (20.64%)
PC1 (49.32%)
Day
d0
d1
d2
d3
d7
d14
d21
C
Relative Abundance
Alcaligenaceae
Bacillaceae
Corynebacteriaceae
Enterobacteriaceae
Enterococcaceae
Flavobacteriaceae
Lachnospiraceae
Lactobacillaceae
Leuconostocaceae
Moraxellaceae
Morganellaceae
Peptostreptococcales-Tissierellales
Planococcaceae
Ruminococcaceae
Weeksellaceae
D
Relative Abundance
[Ruminococcus]_torques_group
Acinetobacter
Alcaligenes
Amphibacillus
Corynebacterium
Empedobacter
Enterococcus
Escherichia-Shigella
Faecalibacterium
Flavobacterium
Kurthia
Lactobacillus
Proteus
Subdoligranulum
Tissierella
Weissella

Figure S1

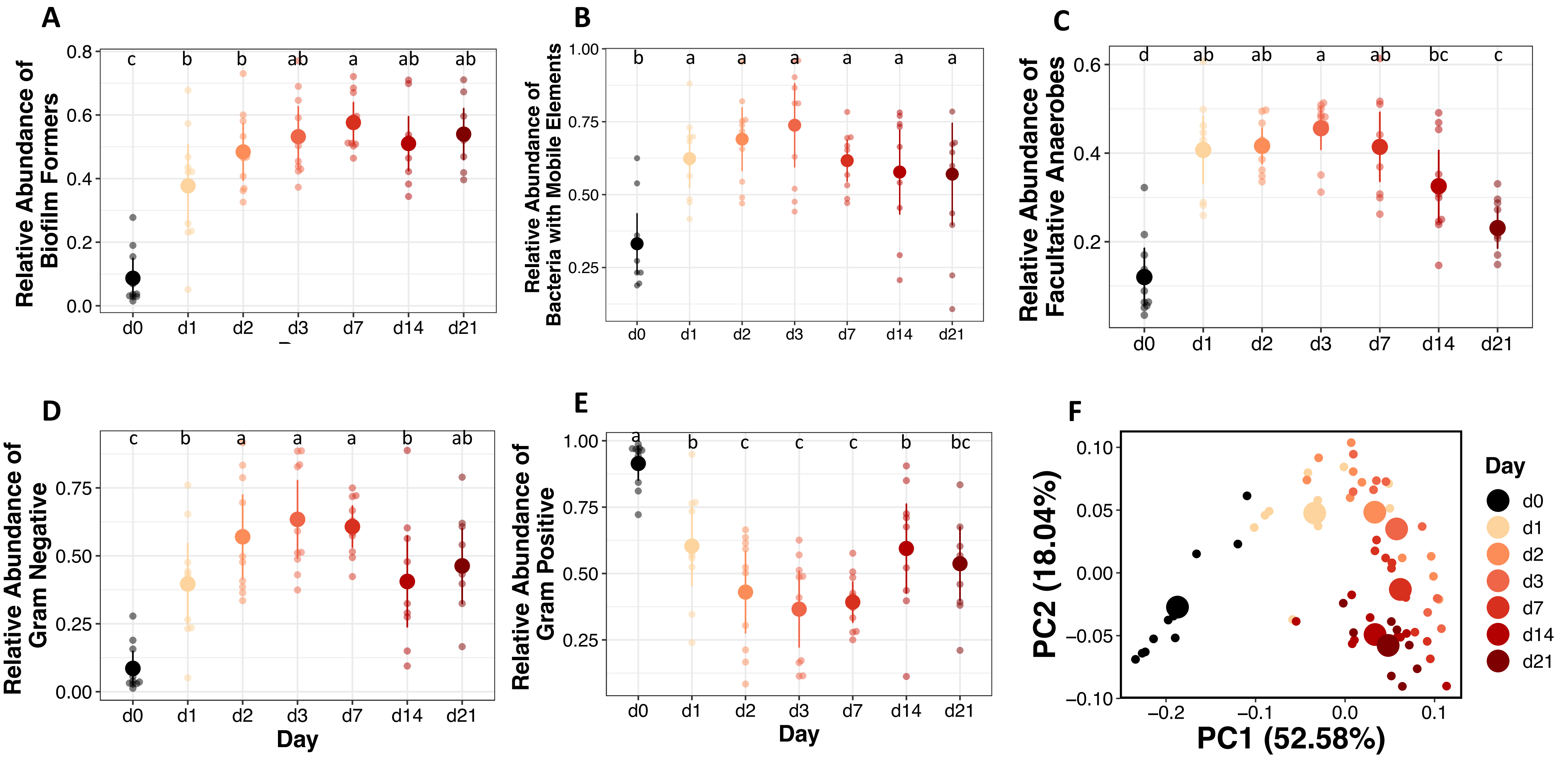
A
Relative Abundance of Biofilm Formers
c b b ab a ab ab
0.0 0.2 0.4 0.6 0.8
d0 d1 d2 d3 d7 d14 d21
B
Relative Abundance of Bacteria with Mobile Elements
b a a a a a a
0.25 0.50 0.75 1.00
d0 d1 d2 d3 d7 d14 d21
C
Relative Abundance of Facultative Anaerobes
d ab ab a ab bc c
0.2 0.4 0.6
d0 d1 d2 d3 d7 d14 d21
D
Relative Abundance of Gram Negative
c b a a a b ab
0.00 0.25 0.50 0.75
d0 d1 d2 d3 d7 d14 d21
Day
E
Relative Abundance of Gram Positive
a b c c c b bc
0.25 0.50 0.75 1.00
d0 d1 d2 d3 d7 d14 d21
Day
F
PC2 (18.04%)
PC1 (52.58%)
−0.10 −0.05 0.00 0.05 0.10
−0.2 −0.1 0.0 0.1
Day
d0
d1
d2
d3
d7
d14
d21

Figure S2

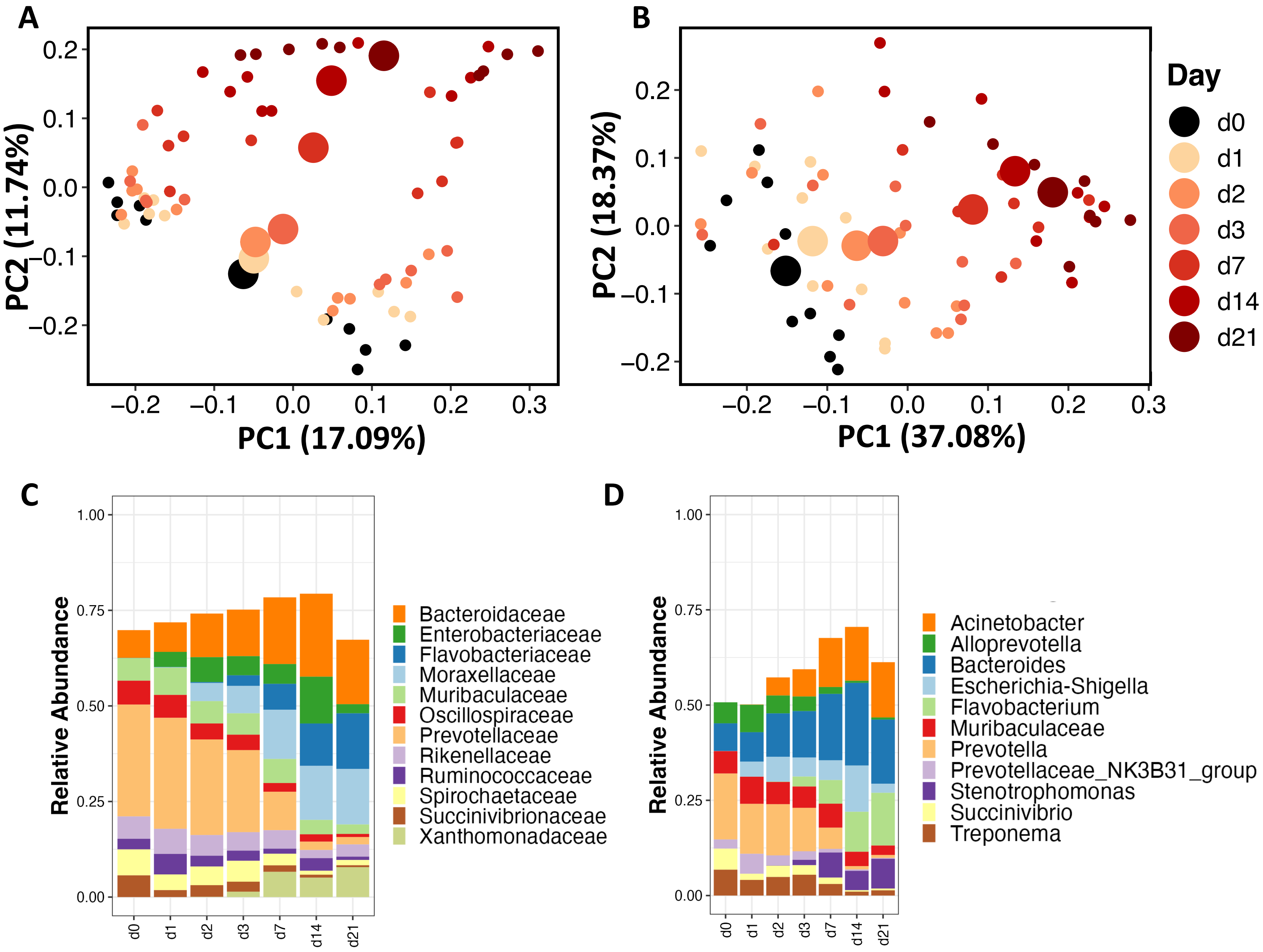
A
PC1 (17.09%)
PC2 (11.74%)
B
PC1 (37.08%)
PC2 (18.37%)
Day
d0
d1
d2
d3
d7
d14
d21
C
Relative Abundance
Bacteroidaceae
Enterobacteriaceae
Flavobacteriaceae
Moraxellaceae
Muribaculaceae
Oscillospiraceae
Prevotellaceae
Rikenellaceae
Ruminococcaceae
Spirochaetaceae
Succinivibrionaceae
Xanthomonadaceae
D
Relative Abundance
Acinetobacter
Alloprevotella
Bacteroides
Escherichia-Shigella
Flavobacterium
Muribaculaceae
Prevotella
Prevotellaceae_NK3B31_group
Stenotrophomonas
Succinivibrio
Treponema

Figure S3

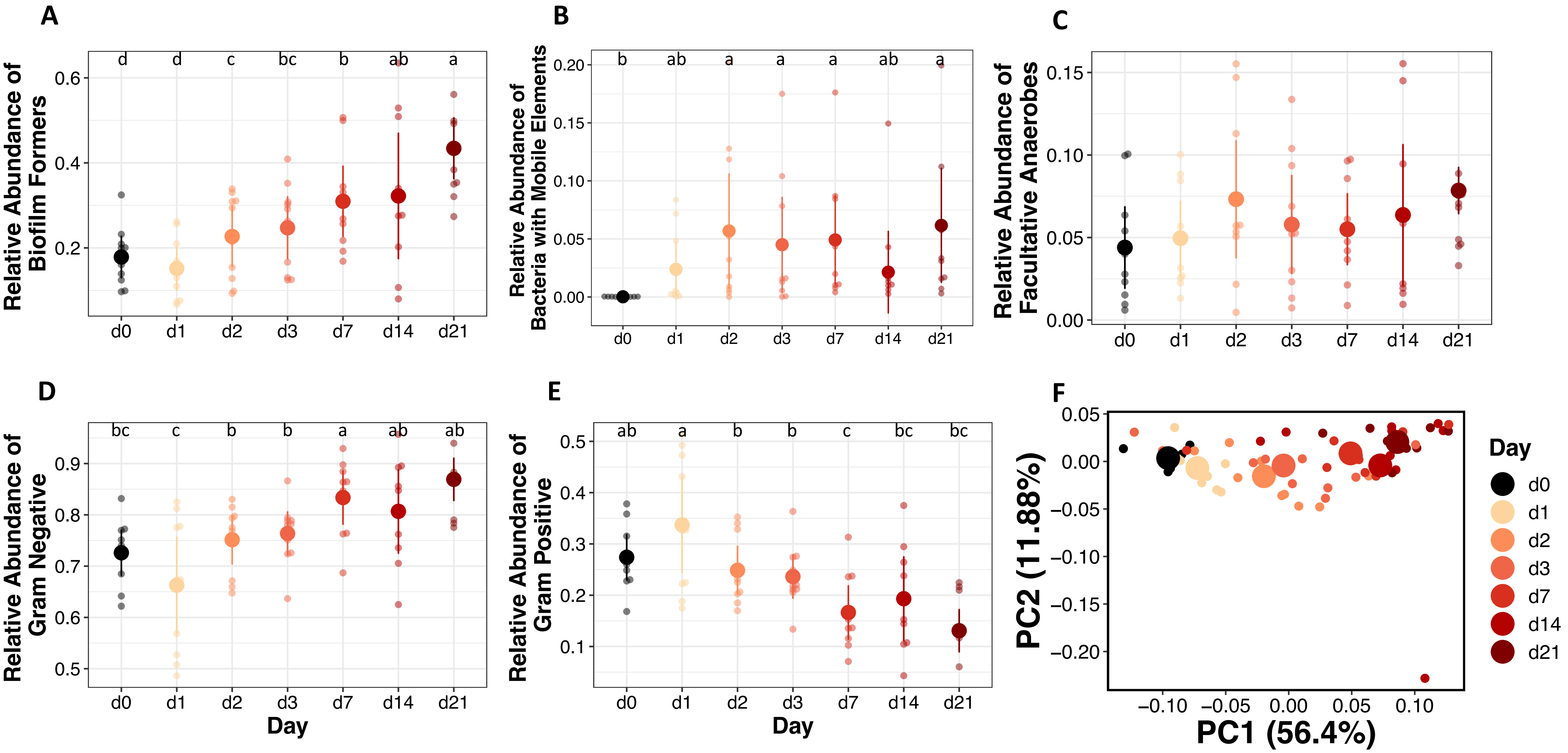
A
Relative Abundance of Biofilm Formers
d d c bc b ab a
B
Relative Abundance of Bacteria with Mobile Elements
b ab a a a ab a
C
Relative Abundance of Facultative Anaerobes
D
Relative Abundance of Gram Negative
bc c b b a ab ab
E
Relative Abundance of Gram Positive
ab a b b c bc bc
F
PC2 (11.88%)
PC1 (56.4%)
Day
d0
d1
d2
d3
d7
d14
d21

Figure S4

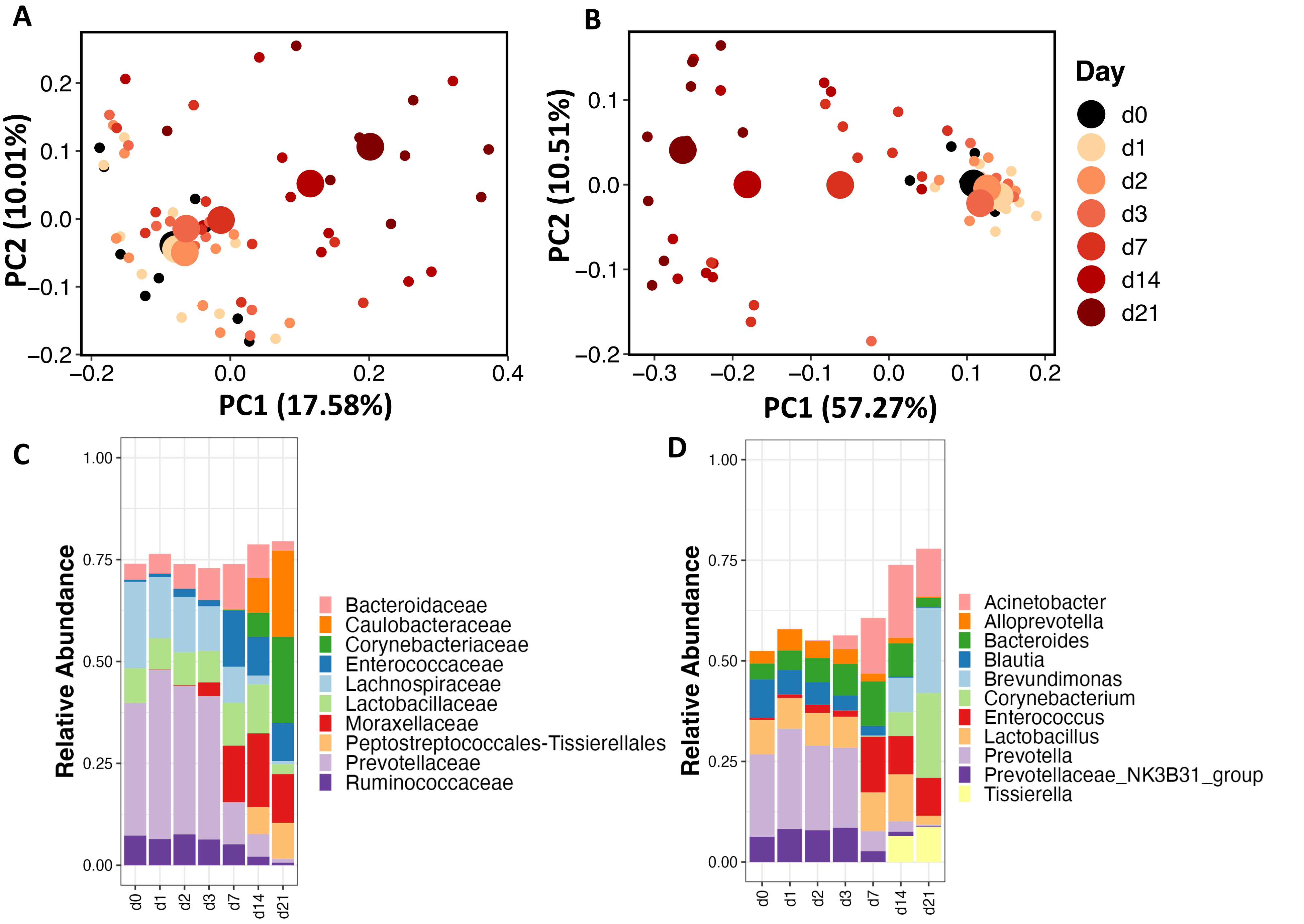
A
B
PC1 (17.58%)
PC2 (10.01%)
PC1 (57.27%)
PC2 (10.51%)
Day
d0
d1
d2
d3
d7
d14
d21
C
D
Relative Abundance
Bacteroidaceae
Caulobacteraceae
Corynebacteriaceae
Enterococcaceae
Lachnospiraceae
Lactobacillaceae
Moraxellaceae
Peptostreptococcales-Tissierellales
Prevotellaceae
Ruminococcaceae
Acinetobacter
Alloprevotella
Bacteroides
Blautia
Brevundimonas
Corynebacterium
Enterococcus
Lactobacillus
Prevotella
Prevotellaceae_NK3B31_group
Tissierella

Figure S5

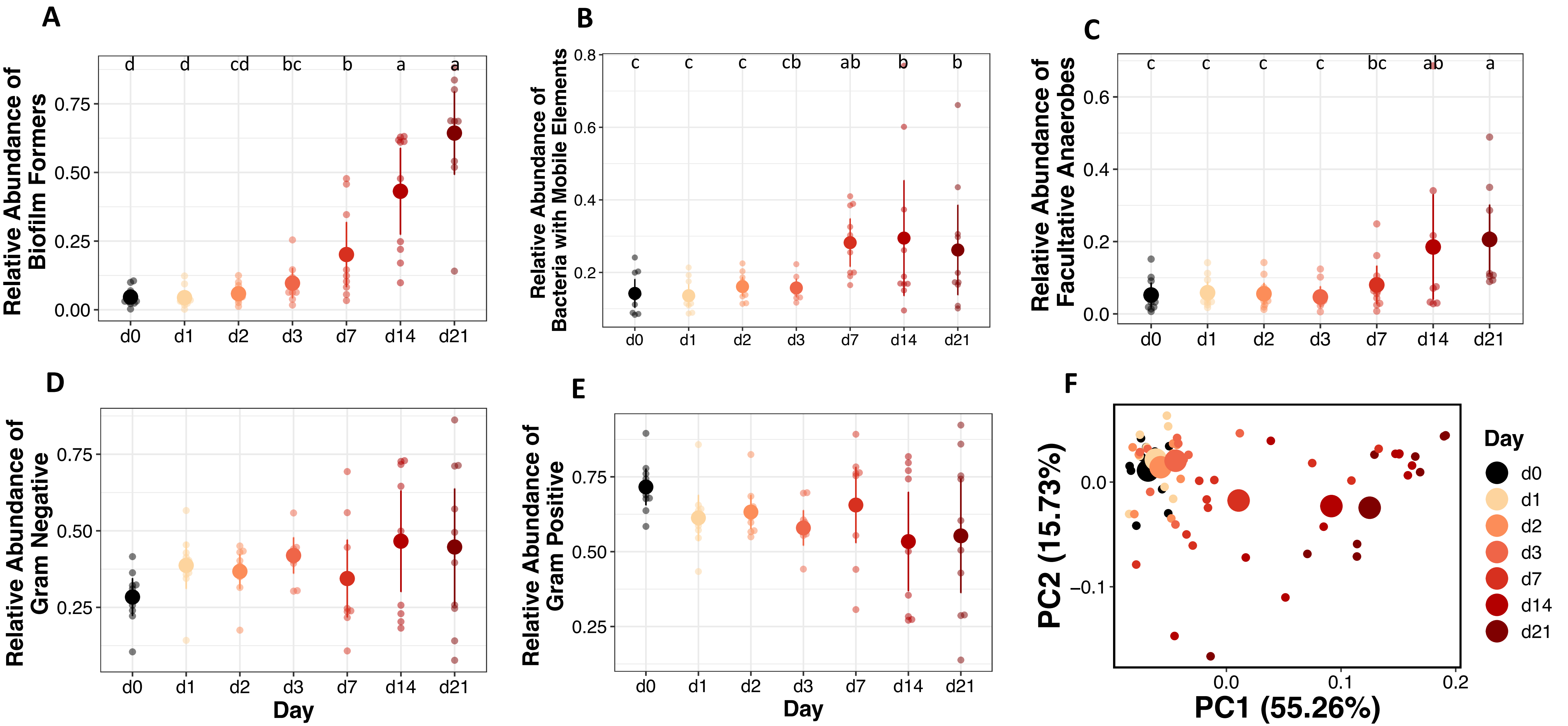
A
Relative Abundance of Biofilm Formers
d d cd bc b a a
0.00 0.25 0.50 0.75
d0 d1 d2 d3 d7 d14 d21
B
Relative Abundance of Bacteria with Mobile Elements
c c c cb ab b b
0.2 0.4 0.6 0.8
d0 d1 d2 d3 d7 d14 d21
C
Relative Abundance of Facultative Anaerobes
c c c c bc ab a
0.0 0.2 0.4 0.6
d0 d1 d2 d3 d7 d14 d21
D
Relative Abundance of Gram Negative
0.25 0.50 0.75
d0 d1 d2 d3 d7 d14 d21
Day
E
Relative Abundance of Gram Positive
0.25 0.50 0.75
d0 d1 d2 d3 d7 d14 d21
Day
F
PC2 (15.73%)
PC1 (55.26%)
0.0 0.1 0.2
0.0 −0.1
Day
d0
d1
d2
d3
d7
d14
d21

Figure S6

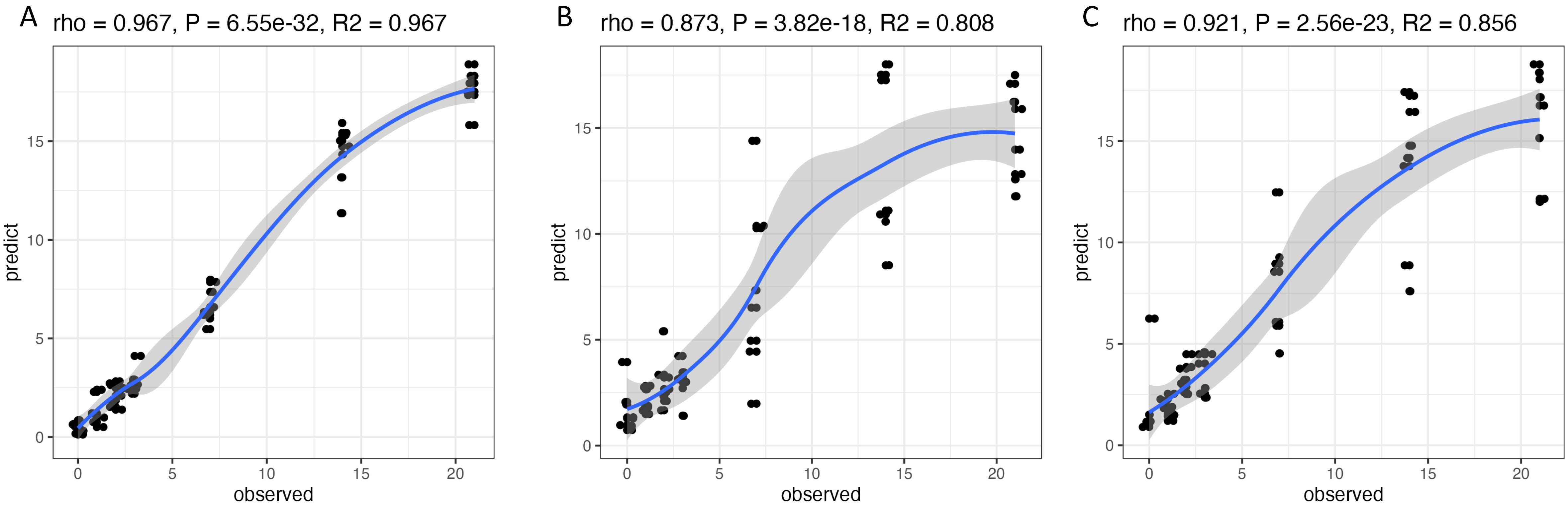
A
rho = 0.967, P = 6.55e-32, R2 = 0.967
predict
observed
B
rho = 0.873, P = 3.82e-18, R2 = 0.808
predict
observed
C
rho = 0.921, P = 2.56e-23, R2 = 0.856
predict
observed

Figure S7
